# Changes in the Demographic Distribution of Chicago Gun-Homicide Decedents From 2015-2021: Violent Death Surveillance Cross-sectional Study

**DOI:** 10.2196/43723

**Published:** 2023-04-07

**Authors:** Maryann Mason, Rushmin Khazanchi, Audrey Brewer, Karen Sheehan, Yingxuan Liu, Lori Post

**Affiliations:** 1 Department of Emergency Medicine Feinberg School of Medicine Northwestern University Chicago, IL United States; 2 Department of Pediatrics, Lurie Children's Hospital of Chicago Feinberg School of Medicine Northwestern University Chicago, IL United States

**Keywords:** gun-homicide surveillance, gun-homicide decedents, demographics, age, gun violence, firearm

## Abstract

**Background:**

Homicide is one of the 5 leading causes of death in the United States for persons aged 1 to 44 years. In 2019, 75% of US homicides were by gun. Chicago has a gun-homicide rate 4 times the national average, and 90% of all homicides are by gun. The public health approach to violence prevention calls for a 4-step process, beginning with defining and monitoring the problem. Insight into the characteristics of gun-homicide decedents can help frame next steps, including identifying risk and protective factors, developing prevention and intervention strategies, and scaling effective responses. Although much is known about gun homicide because it is a long-standing, entrenched public health problem, it is useful to monitor trends to update ongoing prevention efforts.

**Objective:**

This study aimed to use public health surveillance data and methods to describe changes in the race/ethnicity, sex, and age of Chicago gun-homicide decedents from 2015-2021, in the context of year-to-year variation and an overall increase in the city’s gun-homicide rate.

**Methods:**

We calculated the distribution of gun-related homicide deaths by 6 race/ethnicity and sex groups (non-Hispanic Black female, non-Hispanic White female, Hispanic female, non-Hispanic Black male, non-Hispanic White male, and Hispanic male), age in years, and age by age group. We used counts, percentages, and rates per 100,000 persons to describe the distribution of deaths among these demographic groups. Comparisons of means and column proportions with tests of significance set at *P*≤.05 were used to describe changes in the distribution of gun-homicide decedents over time by race-ethnicity-sex and age groups. The comparison of mean age by race-ethnicity-sex group is done using 1-way ANOVA with significance set at *P*≤.05.

**Results:**

The distribution of gun-homicide decedents in Chicago by race/ethnicity and sex groups had been relatively stable from 2015 to 2021 with 2 notable exceptions: a more than doubling of the proportion of gun-homicide decedents who were non-Hispanic Black female (3.6% in 2015 to 8.2% in 2021) and an increase of 3.27 years in the mean age of gun-homicide decedents. The increase in mean age coincided with a decrease in the proportion of non-Hispanic Black male gun-homicide decedents between the ages of 15-19 and 20-24 years and, conversely, an increase in the proportion of non-Hispanic Black male gun-homicide decedents aged 25-34 years<strong>.</strong>

**Conclusions:**

The annual gun-homicide rate in Chicago had been increasing since 2015 with year-to-year variation. Continued monitoring of trends in the demographic makeup of gun-homicide decedents is necessary to provide the most relevant and timely information to help shape violence prevention efforts. We detected several changes that suggest a need for increased outreach and engagement marketed toward non-Hispanic Black female and non-Hispanic Black male individuals between the ages of 25-34 years.

## Introduction

The United States is an outlier among the world’s 28 most populous high-income countries, with a homicide by gun rate 11.4 times that of all these countries combined [1]. Within the United States, gun homicides constitute 75% of all homicides [2] and is one of the 5 leading causes of death for persons aged 1 to 44 years [3]. The US gun homicide rate increased by 34% from 2019 to 2020 [4]. In 2020, Chicago, the third largest US city, had a gun homicide rate of 22.65 per 100,000 persons, nearly 4 times the overall US rate of 5.9 per 100,000 persons [2].

The public health approach to violence prevention calls for a 4-step process, beginning with defining and monitoring the problem [5]. Insight into the characteristics of gun-homicide decedents can help frame next steps, including identifying risk and protective factors, developing prevention and intervention strategies, and scaling effective responses. Although much is known about gun homicide because it is a long-standing, entrenched public health problem, it is useful to monitor trends to update ongoing prevention efforts.

In Chicago, as is the case nationally, the risk for gun-related homicide is not evenly distributed. Adolescents and young adults, males, and Black persons have elevated gun-related homicide rates compared to other population segments [6-8]. Historically, persons between the ages of 15 and 24 years have had the highest gun-related homicide rates [7].

Chicago’s annual rate of gun-related homicides has varied year to year [5,8], but the overall trend has increased since 2015 [9]. In 2021, the number of gun-related homicides in Chicago hit a 40-year high, surpassing the most recent high in 2020 [9]. We wondered if the distribution of gun-homicide decedent demographic characteristics in Chicago had changed with year-to-year variation in Chicago’s gun-homicide rates and in the context of the overall increase in gun-homicide rates.

Understanding changes in decedent characteristics can help advance knowledge of drivers of increases in gun-homicide rates and inform prevention and intervention efforts, including the allocation of resources to prevent future gun homicides. Since prevention settings and content are usually based on intent to reach the populations most at risk, knowledge of changes in the demographics of at-risk populations may help shape more efficacious responses. Consideration of demographic factors such as age and sex in relationship to race/ethnicity is paramount because, as Feld and Bauldry [10] note, gun homicides are unevenly distributed by race/ethnicity and overall summary conclusions may obfuscate important variations.

Our objective was to use public health surveillance data and methods to describe changes in the race/ethnicity, sex, and age of Chicago gun-homicide decedents from 2015-2021, in the context of year-to-year variation and an overall increase in the city’s gun-homicide rate.

## Methods

### Ethical Considerations

This study was determined to be exempt from institutional review board review by the Northwestern University institutional review board because research involving deceased persons is not considered human subjects research, and the data are publicly available and deidentified. The study data source was deidentified retrospective case-level surveillance data from the Cook County Medical Examiner Case Archive [9]. These data were collected after death was declared; therefore, no informed consent was possible or required. To protect against possible identification of individuals in the data set, we suppress counts for cells with fewer than 5 in table presentations.

### Data Sources

Gun-homicide decedent data were from Cook County Medical Examiner Case Archive data [9]. The archive includes data on decedent characteristics and fatal incident date and location. Population data came from American Community Survey [11].

### Inclusion Criteria

This study included all homicides in which the fatal injury occurred in the city of Chicago with the death occurring between January 1, 2015, and December 31, 2021, where the manner of death was ruled “homicide” and the “gun-related” variable was endorsed by the Cook County Coroner’s Office. The start date is January 1, 2015, because that is the first year that electronic surveillance of these deaths had complete case ascertainment.

For our analyses, we used a continuous variable for age in years, as well as a categorical variable where we collapsed the decedent age in years into the following age groups: <15, 15-19, 20-24, 25-34, 35-44, 45-54, 55-64, and 65+ years. Age groups were based on standard age groups generated from the US Census. We also created a race-ethnicity-sex group variable by assigning decedents into 1 of the following 6 race/ethnicity and sex groups: non-Hispanic Black female, non-Hispanic White female, Hispanic female, non-Hispanic Black male, non-Hispanic White male, and Hispanic male. We did not include data on Asian, Native Hawaiian or Other Pacific Islander, or American Indian or Alaska Native racial groups due to the low numbers of decedents, which makes comparisons of distributions unreliable.

### Statistical Analysis

We calculate the overall Chicago annual gun-homicide rate per 100,000 persons as the annual number of gun-homicides divided by the 2020 Chicago population. For analyses, we assigned decedents to 1 of 6 race-ethnicity-sex groups (non-Hispanic Black female, non-Hispanic White female, Hispanic female, non-Hispanic Black male, non-Hispanic White male, and Hispanic male). We graphed the mean age of gun homicide decedents by the year of death and used Pearson *r* to evaluate the association between mean age and the year of death. We described the proportion of decedents by race-ethnicity-sex and age groups over time and tested for statistically significant change over time using comparisons of column proportions with significance set at *P*≤.05. The comparison of mean age by race-ethnicity-sex group was done using 1-way ANOVA with significance set at *P*≤.05. We also examined changes in annual gun-homicide rates per 100,000 persons by age group using the annual number of gun-homicide deaths by age group divided by the annual Chicago population for that age. Analyses were done in IBM Statistics 27. We used graphing procedures in Microsoft Excel 2016, IBM Statistics 27, and Tableau (Salesforce Inc) for data visualizations.

## Results

**Figure 1:**
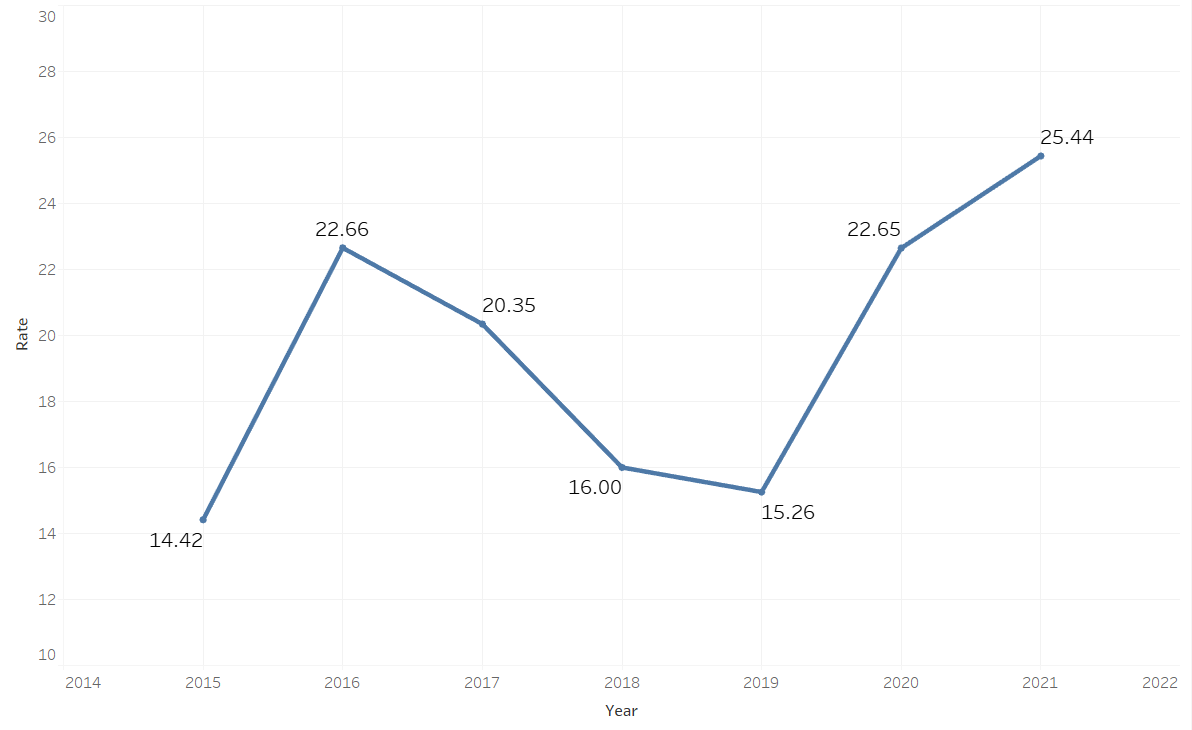
Chicago gun-homicide rate per 100,000 persons: 2015-2021.

Between 2015 and 2021, there were 3723 gun-homicides among Chicago residents recorded by the Cook County Medical Examiner’s Office. Decedents were 92% (n=3425/3723) male, 82% (n=3054/3723) Black, and 15.2% (n=566/3723) Hispanic with a mean age of 28.99 (SD 11.18) years. The overall Chicago gun homicide rate increased 58% from 14.46 to 22.92 per 100,000 persons during this time frame (see Figure 1).

Table 1 reports the annual percentage of gun-homicide deaths by 6 race-ethnicity-sex group combinations. Each row represents the proportion of gun-homicide decedents by race-ethnicity-sex group. Across all years, non-Hispanic Black male individuals consistently made up the largest proportion of gun-homicide decedents in Chicago (≥72.5%). The proportion of gun-homicide deaths among non-Hispanic Black female individuals had more than doubled from 2015 (n=14/391, 3.6%) to 2021 (n=56/685, 8.2%); however, this increase was not statistically significant (*P*>.05).

**Figure 2:**
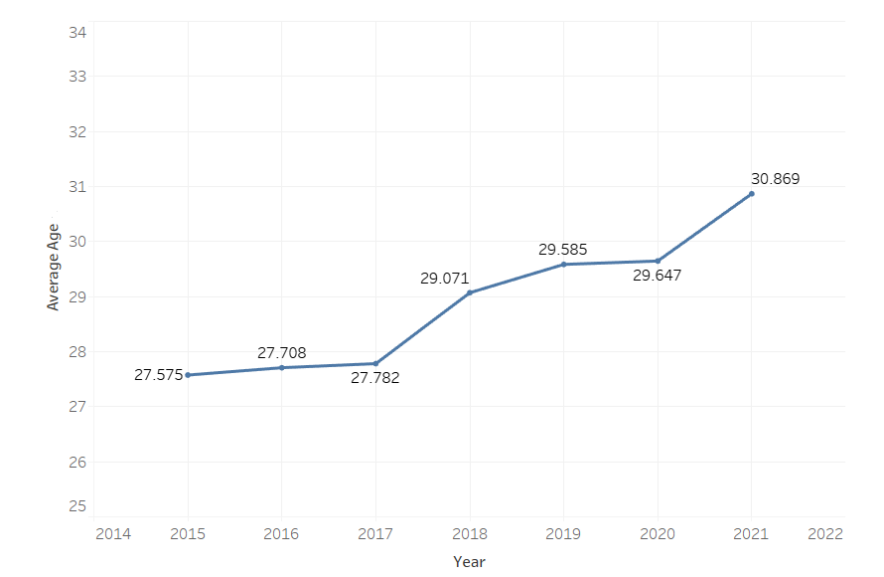
Chicago gun-homicide decedent mean age by year of death: 2015-2021.

From 2015 to 2021, the mean age of gun-related homicide decedents increased by 3.27 years (Figure 2). The largest increase (+1.94 years) occurred between 2017 and 2018.

The mean age among Chicago gun-homicide decedents differed by race-ethnicity-sex group (*F*_5_=9.39; *P*<.001; Table 2). Non-Hispanic White male and female groups had higher mean ages compared to other groups.

**Table 1:** Proportion of Chicago gun-homicide deaths by race-ethnicity-sex groups from 2015-2021.

Race-ethnicity-sex group	Year of death and the proportion of gun-homicide death^a^						
	2015, n (%)	2016, n (%)	2017, n (%)	2018, n (%)	2019, n (%)	2020, n (%)	2021, n (%)
Non-Hispanic Black female	14 (3.6)	27 (4.4)	33 (6)	30 (6.9)	33 (8)	45 (7.3)	56 (8.2)
Non-Hispanic White female	0 (0)	<5 (0.5)	<5 (0.4)	<5 (0.7)	<5 (0.7)	<5 (0.3)	<5 (0.6)
Hispanic female	<5 (0.8)	<5 (1)	6 (1.1)	6 (1.4)	6 (1.5)	<5 (0.6)	10 (1.5)
Non-Hispanic Black male	313 (80.1)	467 (75.9)	409 (74.4)	336 (77.6)	308 (75.1)	448 (72.5)	521 (76.1)
Non-Hispanic White male	8 (2)	14 (2.3)	11 (2)	10 (2.3)	9 (2.2)	16 (2.6)	11 (1.6)
Hispanic male	53 (13.6)	98 (15.9)	89 (16.2)	48 (11.1)	51 (12.4)	103 (16.7)	83 (12.1)

^a^Denominators are not reported due to the suppression of cell counts to protect against possible identification of individuals.

**Table 2:** Chicago gun-homicide decedents’ mean age in years by race-ethnicity-sex group from 2015-2021.

Race-ethnicity-sex group	Year of death and mean age (years)						
	2015	2016	2017	2018	2019	2020	2021
Non-Hispanic Black female	27	28	24	38	28	27	31
Non-Hispanic White female	—^a^	53	60	28	48	31	37
Hispanic female	29	22	24	28	34	24	28
Non-Hispanic Black male	28	28	28	29	29	30	31
Non-Hispanic White male	31	34	34	41	34	30	39
Hispanic male	27	27	27	27	29	30	30

^a^Not applicable.

We also examined the annual distribution of decedents by age group across each of the 6 race-ethnicity-sex groups using tests of column proportions to evaluate statistical significance of differences (data not shown). Only 2 of the 6 race-ethnicity-sex groups examined had statistically significant changes in the annual distribution of decedents. The non-Hispanic Black female group had a statistically significant decrease in the proportion of gun-homicide deaths among those aged 65+ years, dropping from 16.7% (n=5/30) of gun-homicide deaths among non-Hispanic Black female individuals in 2018 to 2.2% (n<5/45) in 2020 (*P*=.02)*.* However, the total number of non-Hispanic Black female deaths among those aged 65+ years during the study period was low (n=6/3702).

Among the non-Hispanic Black male group, there were statistically significant drops in the proportion of 15–19-year old gun-homicide decedents from 2016 (n=94/615, 20.1%) to 2020 (n=53/618, 11.8%; *P*=.01), from 2016 (n=94/615, 20.1%) to 2021 (n=54/685, 10.4%; *P*<.001), and from 2017 (n=78/550, 19.1%) to 2021 (n=54/685, 10.4%; *P*=.003). Among the non-Hispanic Black male group, there were statistically significant drops in the proportion of 20–24-year-old gun-homicide decedents from 2015 (n=95/391, 30.4%) to 2020 (n=81/618, 18.1%; *P*=.002), from 2015 (n=95/391, 30.4%) to 2021 (n=104/685, 20%; *P*=.01), and from 2016 (n=125/615, 26.8%) to 2020 (n=81/618, 18.1%; *P*=.04). Meanwhile, there were statistically significant increases in the proportion of 25–34-year-old non-Hispanic Black male gun-homicide decedents from 2015 (n=99/391, 31.6%) to 2020 (n=191/618, 42.6%; *P*=.04) and from 2016 (n=146/615, 31.3%) to 2020 (n=191/618, 42.6%; *P*=.008).

**Figure 3:**
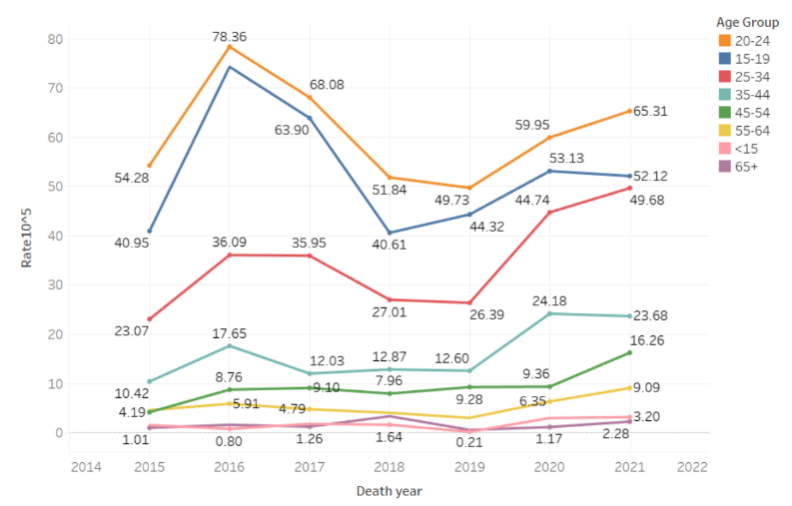
Chicago gun-homicide rate by age group from 2015-2021.

We also examined gun-homicide rates per 100,000 persons for each age group (see Figure 3). Rates offer important information about the relative risk to groups based on their proportion of the total population. The highest gun-homicide rates were consistently among the 15-19, 20-24, and 25-34 years age groups. From 2020 to 2021, the 15-19 and 35-44 years age groups saw slight declines. Most other groups increased, although the magnitude of increase differed by age group.

## Discussion

### Principal Findings

The annual distribution of gun-homicide decedents by race-ethnicity-sex group had been relatively stable over time, even in the context of year-to-year changes in gun-homicide rates and the 2015-2021 increase in gun-homicide rates in Chicago. There was a large but nonstatistically significant increase in the proportion of non-Hispanic Black female gun-homicide decedents from 2015 (3.6%) to 2021 (8.2%). The lack of statistical significance may be due to a relatively small (238/3723, 6.4%) number of non-Hispanic Black female individuals among gun-homicide decedents. The mean age of Chicago gun-homicide decedents increased by 3.27 years from 2015-2021. This increase in mean age coincided with a decrease in the proportion of non-Hispanic Black male gun-homicide decedents between the ages of 15-19 and 20-24 years and, conversely, an increase in the proportion of non-Hispanic Black male gun-homicide decedents between the ages of 25-34 years.

Although there is much discussion of firearm violence in Chicago in news publications [12] and, less often, research literature [13,14], there is little detailed information available on the demographics of gun-homicide decedents in the face of changes in violence other than to note broad populations at the highest risk at one point in time. This study examined the intersection of race/ethnicity, sex, and age among of Chicago gun-homicide decedents over a 7-year period in which there were year-to-year changes in overall gun-homicide rates. It provides key information on group representation in gun homicide during periods of escalation and periods of retraction. The changes in the makeup of gun-homicide decedents we detected signal a need for expanded prevention outreach and intervention engagement marketed toward non-Hispanic Black female individuals of all ages and non-Hispanic Black male individuals between the ages of 25-34 years, while at the same time, efforts are needed to expand outreach to and engagement of younger non-Hispanic Black male individuals, who consistently have the highest gun-homicide rates in Chicago.

This study has several strengths and limitations. In terms of strengths, the study incorporated very recent and reliable surveillance data from the Cook County Medical Examiner’s Office, a nonpartisan office led by an appointed chief medical examiner. However, it is well known that death certificate reporting of race and ethnicity underreports certain subgroups [15], and so, it is likely that certain race/ethnicity groups, especially Alaska Native or American Indian individuals, those with more than one race/ethnicity affiliation, and Hispanic individuals may be underreported in the Medical Examiner data used in this study. Regarding other limitations, our reliance on US Census age groups as denominators for calculating homicide rates prevents a more nuanced analysis of single-year age risks. Further, although we report on demographics of gun-homicide decedents, ideally, more information on the circumstances surrounding these gun-related homicide deaths is needed to inform intervention approaches.

### Conclusions

The findings from this modest study provide a basis for informing firearm violence prevention and intervention strategies and can inform a research agenda going forward. Further research could address the following questions: Will this shift toward increased non-Hispanic Black female and non-Hispanic Black male gun-homicide decedents between the ages of 25-34 year endure over time? What are the drivers associated with this increase in the proportion of non-Hispanic Black female gun-homicide decedents? and Are these age and gender shifts accompanied by changes in the precipitators and features of gun-homicide events? Because gun-homicide is a leading public health problem in the United States and, even more so, locally in Chicago, understanding the stability of and changes within decedent demographic subgroups is part of a required first step to inform targeted prevention responses.

## Data Availability

Gun-homicide decedent data are available from the Cook County Medical Examiner Case Archive [9]. Population data came from the 2020 US Census [11].
